# Vaginal surgery for pelvic organ prolapse using mesh and a vaginal support device

**DOI:** 10.1111/j.1471-0528.2007.01606.x

**Published:** 2008-02

**Authors:** M Carey, M Slack, P Higgs, M Wynn-Williams, A Cornish

**Affiliations:** a Department of Urogynaecology, Royal Women’s Hospital Melbourne, Victoria, Australia; b Department of Urogynaecology, Addenbrooke’s Hospitals Cambridge, UK

**Keywords:** Mesh, prolapse, vaginal support device

## Abstract

**Objectives:**

To describe a new surgical procedure for pelvic organ prolapse using mesh and a vaginal support device (VSD) and to report the results of surgery.

**Design:**

A prospective observational study

**Setting:**

Two tertiary referral Urogynaecology practices.

**Population:**

Ninety-five women with International Continence Society pelvic organ prolapse quantification stage 2 or more pelvic organ prolapse who underwent vaginal surgery using mesh augmentation and a VSD.

**Methods:**

Surgery involved a vaginal approach with mesh reinforcement and placement of a VSD for 4 weeks. At 6 and 12 months, women were examined for prolapse recurrence, and visual analogue scales for satisfaction were completed. Women completed quality-of-life (QOL) questionnaires preoperatively and at 6 and 12 months.

**Main outcome measures:**

Objective success of surgery at 6 and 12 months following surgery. Secondary outcomes were subjective success, complications, QOL outcomes and patients’ satisfaction.

**Results:**

Objective success rate was 92 and 85% at 6 and 12 months, respectively. Subjective success rate was 91 and 87% at 6 and 12 months, respectively. New prolapse in nonrepaired compartments accounted for 7 of 12 (58%) failures at 12 months. Two of 4 mesh exposures required surgery. Sexual dysfunction was reported by 58% of sexually active women preoperatively and 23% at 12 months. QOL scores significantly improved at 12 months compared with baseline (*P* < 0.0001).

**Conclusion:**

Vaginal surgery using mesh and a VSD is an effective procedure for pelvic organ prolapse. However, further studies are required to establish the role of the surgery described in this study.

*Please cite this paper as:*Carey M, Slack M, Higgs P, Wynn-Williams M, Cornish A. Vaginal surgery for pelvic organ prolapse using mesh and a vaginal support device. BJOG 2008;115:391–397.

## Introduction

Many different vaginal, abdominal and laparoscopic procedures have been described to treat pelvic organ prolapse. There is no consensus on the most effective operation. Each year in the USA, approximately 200 000 women undergo surgery for pelvic organ prolapse.^1^ In a large study from a US health maintenance organisation, it was reported that 11.1% of women had undergone surgery for pelvic organ prolapse or urinary incontinence or both by the age of 80 years.^2^ Repeat surgery for recurrent prolapse was required in 29.2% within 4 years of the primary surgical procedure. Risk factors for recurrent prolapse are poorly understood, but it appears that women aged less than 60 years and women with higher grades of prolapse (International Continence Society pelvic organ prolapse classification [ICS POP-Q] stages 3 and 4) are more likely to experience recurrent prolapse after vaginal repair surgery.^3^,^4^

The high rate of failure with conventional colporrhaphy for pelvic organ prolapse has led to an increasing use of synthetic and biological grafts to augment vaginal repair procedures to obtain more durable results. This approach employs the interposition of a prosthesis (synthetic, autologous, allograft or xenograft) between vaginal epithelium and the underlying fascia. Significant problems associated with the use of mesh during vaginal surgery for pelvic organ prolapse have been reported and include dyspareunia and mesh exposure.^5^

This paper describes a new approach to the surgical management of pelvic organ prolapse. Surgery involves a simple and novel approach using mesh and placement of a vaginal support device (VSD) into the lumen of the vagina at the completion of surgery. In this study, careful attention was paid to mesh handling and closure of the vaginal epithelium over the mesh. The aim of this study was to describe this new surgical procedure and to report the results of surgery.

## Patients and methods

Between June 2004 and February 2005, all eligible women with POP-Q stage 2 or more at the anterior and/or posterior vaginal sites were included in this study. Women were assessed clinically, and the prolapse was staged using the ICS POP-Q classification system.^4^ Multichannel urodynamics was performed prior to surgery on women with urinary incontinence. Women completed a prolapse-specific validated questionnaire (Prolapse Symptom Inventory and quality-of-life questionnaire [PSI-QOL]) prior to surgery.^6^,^7^ For the PSI-QOL questionnaire, comparison between baseline and 12-month review data was analysed using a paired *t* test. The women also completed a visual analogue scale (VAS) of their bother with prolapse. Consent for surgery was obtained from all women. This study was considered a clinical surgical audit, and formal institutional review board approval was not required. At the Melbourne site, clinical ethical committee approval was obtained to perform this surgery.

**Figure 1:**
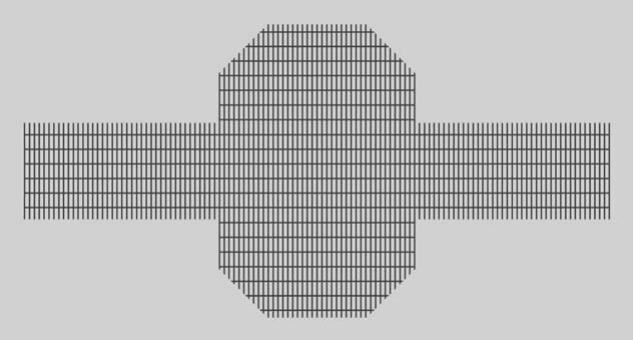
Cross-shaped mesh used for anterior vaginal repair.

**Figure 2:**
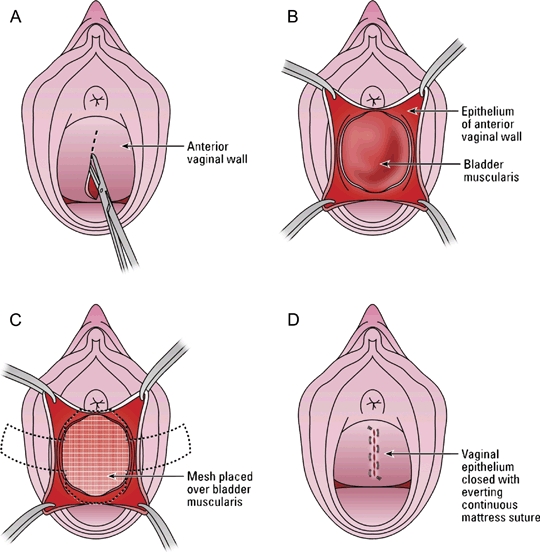
(A–D) Anterior vaginal repair using mesh augmentation. (A) Vaginal incision. (B) Epithelium is dissected off the underlying prevesical tissue. (C) Mesh is placed over the prevesical tissue. (D) Epithelium is closed in two layers.

The surgical technique varied according the site of the pelvic organ prolapse. Gynemesh PS mesh (Ethicon, Somerville, NJ, USA) was used during surgery for all cases. When mesh was used in the anterior vaginal repair, the vaginal epithelium was dissected off the underlying prevesical tissue. Laterally, dissection continued until each arcus tendineus faciae pelvis was reached. Lateral dissection was continued into right and left paravaginal spaces so that the inner aspect of the pubic bone could be palpated. A modified repair of the prevesical tissue was performed using 2/0 Monocryl (Ethicon). Only the central part of the prevesical tissue was repaired. This avoided narrowing of the prevesical space. A cross-shaped piece of mesh was cut and placed over the prevesical tissue with the extension arms being placed into each paravaginal space (Figure 1). The mesh abutted the inner aspect of the pubic bone on each side (Figure 2).

**Figure 3:**
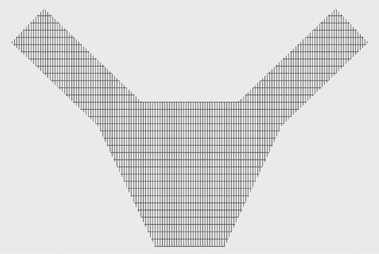
‘Y’-shaped mesh used for posterior vaginal repair.

**Figure 4:**
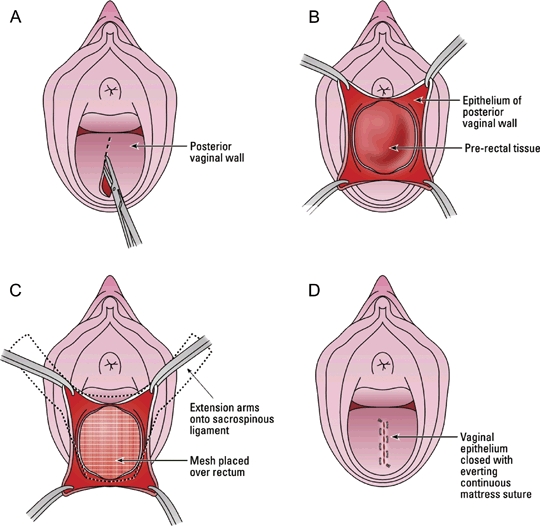
(A–D) Posterior vaginal repair with mesh augmentation. (A) Vaginal incision. (B) Epithelium is dissected off the underlying prerectal tissue. (C) Mesh is placed over the prerectal tissue and onto each sacrospinous ligament. (D) Epithelium is closed in two layers.

When mesh was used to reinforce the posterior vaginal repair, the vaginal epithelium was dissected off the underlying prerectal tissue. Dissection continued laterally on each side to the levator ani muscles. The dissection continued in a superior and lateral direction through the rectal pillars to each ischial spine and sacrospinous ligament. Only the central part of the prerectal tissue was repaired. This modification was undertaken to avoid narrowing of the prerectal space. A ‘Y’-shaped piece of mesh was placed over the prerectal tissue with the extension arms of the mesh being placed in the tunnels created by the dissection onto the sacrospinous ligaments (Figure 3). The mesh abutted each sacrospinous ligament. Sutures were not placed into the sacrospinous ligaments, thus reducing the amount of dissection that is usually required to perform a sacrospinous colpopexy. Mesh was not placed in the lower third of the posterior vaginal wall. At this level, the vaginal epithelium is fused laterally to the levator ani muscles and posteriorly to the perineal body (Figure 4).

When mesh was used to reinforce both anterior and posterior vaginal walls, separate pieces of mesh were placed, in turn, under the anterior and posterior vaginal wall epithelium as described above.

In the presence of a cystocele and vault prolapse but with no rectocele, the placement of the mesh under the anterior vaginal wall epithelium differed from the technique described above. The anterior vaginal wall epithelium was dissected off the underlying prevesical tissue. Laterally, dissection continued until each arcus tendineus faciae pelvis was reached. Dissection continued in a superior and lateral direction until each sacrospinous ligament was reached. A ‘Y’-shaped piece of mesh was placed over the prevesical tissue with the extension arms of the mesh being placed in the tunnels created by the dissection onto the sacrospinous ligaments. Sutures were not placed in the sacrospinous ligaments.

During surgery, the vaginal wounds were irrigated with saline. The mesh was soaked in an antibiotic solution before vaginal insertion. Handling of the mesh was kept to a minimum, and cutting of the mesh was performed using clean scissors.

Any trimming of excess vaginal epithelium was kept to a minimum. The vaginal epithelium was closed in two layers. The deeper layer was closed using a continuous subepithelial noninterlocking stitch of 2/0 Monocryl. The epithelium was then closed by a noninterlocking continuous everting mattress stitch using 2/0 Vicryl (Ethicon). A noninterlocking stitch was used to avoid devascularising of the vaginal epithelium along the incision lines and may reduce mesh exposure. The two-layered closure, including the everting mattress stitch, was used to obtain a relatively thick suture line at the site of the vaginal incision.

All women received intravenous prophylactic antibiotic therapy that was continued for 48 hours following surgery followed by 5 days of oral antibiotic therapy. Enoxaparin was routinely used in each woman and continued until the woman was discharged from hospital.

**Figure 5:**
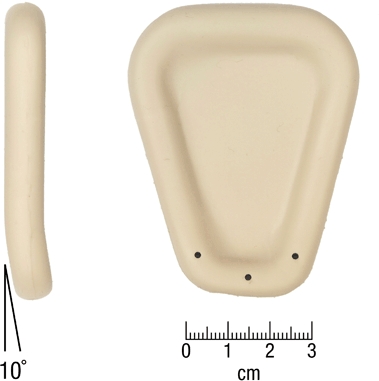
Side and front views of medium-sized VSD.

At the completion of surgery, an appropriately sized VSD was placed in the vagina and sutured in place with 2/0 Monocryl to prevent dislodgement. The VSD is made of medical grade silicon and available in three sizes (Figure 5). After the woman was discharged home, the first review was at 4 weeks to remove the VSD in the consulting room. By 4 weeks, the sutures holding the VSD in place had dissolved.

Further reviews were at 6 and 12 months. At 6 and 12 months, women underwent clinical evaluation and completed a VAS detailing their satisfaction with surgery. The 12-month POP-Q examination was performed by a nonsurgical author or in the presence of a nonsurgical author when undertaken by a surgical author. Women completed the PSI-QOL questionnaire at 6 and 12 months. Questionnaires were handed to, or posted to, women by a research nurse at each centre and were completed in private by the women. Success or failure was determined objectively (success: stages 0 and 1 at all sites, failure: stage 2 or more prolapse at any site), subjectively (symptom of vaginal pressure, success: ‘never or rarely’; failure: ‘sometimes, most times or all times’) and by VAS (success: VAS 8 or more, failure: VAS <8).

## Results

Ninety-five women underwent surgery for pelvic organ prolapse at two centres (Melbourne, Australia and Cambridge, UK). The Melbourne site contributed 84 cases, and 11 cases were from the Cambridge site. The mean age of the women was 59 years (SD ±10 years) and mean parity of 2.6 (SD ±1.1). Forty women (43%) had undergone a prior hysterectomy and 23 (24%) had at least one surgical procedure for pelvic organ prolapse. Thirteen women (14%) had undergone surgery for stress incontinence. A total of 31 (33%) women had undergone prior surgery for pelvic organ prolapse and/or stress urinary incontinence (Table 1).

**Table 1:** Patient demographics and details of previous pelvic operations

Variables	Result
**Mean age (SD)**	59.2 (±10.1)
**Mean BMI (SD)**	29.4 (±6.1)
**Mean parity (SD)**	2.7 (±1.1)
**Previous surgery**	***n* (%)**
Hysterectomy	40 (42.1)
Vaginal repair	23* (24.2)
Continence surgery	13* (13.7)

BMI, body mass index.

* Some women had multiple prior surgical procedures.

The operations performed on the 95 women are detailed in Table 2. Mesh was used for the anterior vaginal repair only in 6 (6%) women, posterior vaginal repair only in 26 (27%) women and both anterior and posterior repairs in 63 (66%) women. Of the 55 women who had not undergone a prior hysterectomy, a vaginal hysterectomy was performed in 29 women and the uterus was retained in 26 women. In some cases, concomitant surgical procedures were undertaken (Table 2).

**Table 2:** Details of surgery performed for pelvic organ prolapse

Surgery	*n* (%)
**Surgery using mesh**	
Anterior repair	6 (6.3)
Posterior repair	26 (27.4)
Anterior and posterior repair with mesh	63 (66.3)
Concomitant surgical procedures*	
TVT	1 (1.1)
TVT-O	31 (32.6)
Vaginal hysterectomy	29 (30.5)
Vaginoplasty for vaginal stenosis	3 (3.2)
Anal sphincter repair	1 (1.1)

TVT, tension-free vaginal tape; TVT-O, tension-free vaginal tape obturator.

* Some women underwent multiple procedures.

At 6 months, 78 (82.1%) women returned for physical examination and 80 (84.2%) at 12 months. QOL questionnaires and VAS for satisfaction with surgery were completed by 74 (77.9%) women at 6 months and 84 (88.4%) at 12 months. The POP-Q outcomes by vaginal compartment are detailed in Table 3. Twelve (15%) women have developed further objective (ICS POP-Q stage 2 or more) pelvic organ prolapse. Seven of these 12 prolapses were de novo with the prolapse occurring in the compartment not surgically repaired. Significantly more failures occurred in women when an anterior or posterior compartment was not repaired compared with women having both anterior and posterior compartments repaired (*P* = 0.0168 Fisher’s exact test). Assuming the 15 women not examined at 12 months were objective failures, then the failure rate would be 28.4%. Whereas, assuming the 15 women not examined at 12 months were objective successes, then the failure rate would be 12.6%.

**Table 3:** Overall and compartment stages of pelvic organ prolapse at baseline, 6 and 12 months

POP-Q stages	Baseline (*n* = 95), *n* (%)	6 months (*n* = 98), *n* (%)	12 months (*n* = 80), *n* (%)
**Overall POP-Q**			
Stage 0	0 (0)	44 (56.4)	33 (41.2)
Stage 1	0 (0)	28 (35.9)	35 (43.8)
Stage 2	68 (71.6)	6 (7.7)	11 (13.8)
Stage 3	24 (25.3)	0 (0)	1 (1.2)
Stage 4	3 (3.2)	0 (0)	0 (0)
**Anterior POP-Q**			
Stage 0	9 (9.5)	53 (68)	44 (55)
Stage 1	19 (20)	20 (25.6)	28 (35)
Stage 2	57 (60)	5 (6.4)	7 (8.8)
Stage 3	10 (10.5)	0 (0)	1 (1.3)
Stage 4	0 (0)	0 (0)	0 (0)
**Posterior POP-Q**			
Stage 0	3 (3.2)	70 (89.7)	64 (80)
Stage 1	9 (9.5)	7 (9)	11 (13.8)
Stage 2	71 (74.7)	1 (1.3)	5 (6.3)
Stage 3	12 (12.6)	0 (0)	0 (0)
Stage 4	0 (0)	0 (0)	0 (0)
**Vault/cervix POP-Q**			
Stage 0	23 (24.2)	74 (94.9)	78 (97.5)
Stage 1	47 (49.5)	4 (5.1)	2 (2.5)
Stage 2	13 (13.7)	0 (0)	0 (0)
Stage 3	9 (9.5)	0 (0)	0 (0)
Stage 4	3 (3.2)	0 (0)	0 (0)

The anterior compartment was the most common site for recurrent prolapse (eight cases), followed by posterior compartment (five cases). One woman had recurrent prolapse of both anterior and posterior compartments. There was no case of recurrent prolapse at the apical compartment. Of the six women who underwent repair of the anterior compartment, two (33%) developed de novo prolapse of the posterior compartment. Of the 26 women who underwent repair of the posterior compartment, 23 were examined at 12 months, and 5 (22%) had de novo prolapse of the anterior compartment and 1 (4%) had a recurrent rectocele.

The summary results of surgery, satisfaction with surgery and QOL questionnaires at the 6 and 12 months are detailed in Table 4. Women reporting vaginal pressure symptoms ‘sometimes, most times or all times’ reduced from 84 of 93 (90%) preoperatively to 7 of 75 (9%) and 11 of 84 (13%) at 6 and 12 months.

**Table 4:** Summary of surgical, satisfaction with surgery and QOL outcomes

Outcome measures	Preoperative	6 months	12 months	
Objective success (ICS POP-Q stages 0 and 1)	0/95 (0%)	72/78 (92%)	68/80 (85%)	
	*n* = 95	*n* = 75	*n* = 84	*P* value
Mean VAS for satisfaction with surgery (SD)	–	8.3 (±2.0)	8.2 (±2.1)	
Mean PSI (SD)	2.48 (±0.52)	1.50 (±0.39)	1.58 (±0.42)	<0.0001*
Mean QOL (SD)	2.03 (±0.80)	1.31 (±0.59)	1.34 (±0.57)	<0.0001*

* *P* values are for comparisons between preoperative and 12-month scores.

At 12 months, the average VAS score was 8.2 (SD ± 2.1) with 26 (31%) of 83 women reporting a VAS of <8 and 57 (69%) reporting a score of 8 or more. Sexual dysfunction was reported by 58% (47 of 81) women preoperatively. This reduced to 20% (13 of 64) and 23% (18 of 78) at 6 and 12 months, respectively. There were 77 women with complete preoperative and 12-month PSI-QOL data. The PSI component improved from 2.48 (SD ± 0.52) at baseline to 1.58 (SD ± 0.42) at 12 months (*P* < 0.0001). The QOL domain improved from 2.03 (SD ± 0.80) preoperatively to 1.34 (SD ± 0.57) at 12 months (*P* < 0.0001).

The mean operating time from first incision to placement of the VSD was 59 minutes (SD ± 20 minutes, range 20–120 minutes). No major intraoperative complication occurred. One rectal perforation occurred during surgery. One woman developed a pulmonary embolus inspite of prophylaxis with enoxaparin. This woman was treated with warfarin for 6 months. One woman developed a pelvic haematoma requiring transvaginal drainage.

A small, medium or large VSD was used in 33, 45 and 16 women, respectively. The size of the VSD used in one woman was not recorded. The VSD was generally well tolerated by the women. Awareness of the VSD was reported by 31 women. Discomfort from the VSD was reported as ‘slight’ by 22 women and ‘significant’ by three women. In three women, the VSD was removed at the women’s request before the 4-week review. In one woman, the VSD spontaneously extruded 2 weeks after surgery. VSD ‘protrusion’ was reported by 14 women and ‘slippage’ by 8 women. From 2 to 3 weeks following surgery, some women reported a slight vaginal discharge. This discharge resolved 1–2 days following removal of the VSD.

Postoperative complications are detailed in Table 5. There were four mesh exposures. Three were located in the anterior vaginal wall and one in the posterior vaginal wall. In two women, the mesh exposures were simply treated by transvaginal excision of the eroded mesh after mobilisation of the surrounding vaginal epithelium. In two women, the mesh exposure was treated medically with vaginal estrogen therapy. Sexual dysfunction requiring further surgery was due to a mid-vaginal constriction in three women and a perineal band in one woman.

**Table 5:** Postoperative complications

Complication	*n* (%)
**Mesh exposure**	4 (4.2)
**Stress incontinence**	2 (2.1)
**Obstructive voiding**	1 (1.1)
**Pelvic haematoma**	1 (1.1)
**Further surgery**	
TVT-O	2 (2.1)
Division of TVT-O	1 (1.1)
Vaginoplasty	3 (3.2)
Division of perineal band	1 (1.1)
Excision of exposed mesh	2 (2.1)

## Discussion

Following surgery for pelvic organ prolapse, the repaired tissues are exposed to rises in intra-abdominal pressure as the woman mobilises or with coughing, vomiting and straining with bowel evacuation. Rises in intra-abdominal pressure may adversely effect the healing of the vaginal repair procedure leading to surgical failure and recurrent prolapse. By reinforcing the vaginal repair procedure with mesh and supporting the vagina with the VSD for 4 weeks following surgery, the risk of surgical failure and recurrent pelvic organ prolapse is likely to be reduced. Mesh is incorporated into the body tissues at 3 weeks. The VSD supports not only the vaginal tissues after surgery but also the positioning of the mesh. By supporting the positioning of the mesh until incorporation into the body tissues occurs, it is possible to avoid placing sutures into the sacrospinous ligaments or paravaginal spaces. This procedure avoids need for dissection outside the pelvic cavity. This makes surgery much simpler to perform and reduces the risk of the specific complications that can occur with suture placement into these structures or when tunnelling devices are used beyond the pelvic cavity.^8^–^10^

Prolapse following surgery was often de novo, occurring in the compartment not surgically repaired. De novo prolapse accounted for 58% (7 of 12) of the surgical failures. Failures were significantly more likely to occur when an anterior or posterior compartment was not repaired, and the failure occurred in the nonrepaired compartment in 7 of 8 women. Whether to surgically repair nonprolapsed compartments concomitantly with surgical repair of prolapsed compartments remains an unresolved issue.

Reduction in the prevalence of postoperative sexual dysfunction and the low mesh exposure rate described in this study compared with other studies may be due to the surgical technique.^5^,^11^ In particular, the avoidance of placing mesh in lower third of the posterior vaginal wall, the modifications used during prevesical and prerectal tissues plication, the two-layered technique for closing the vaginal epithelium and the use of a lightweight mesh. Modifications used during plication of the prevesical and prerectal tissues are less likely to compromise vaginal capacity. The two-layered technique for closing the vaginal epithelium without using an interlocking suture is likely to reduce devascularisation and separation of the vaginal epithelium along the suture line over the mesh. The placement of the VSD for 4 weeks following surgery may reduce tissue contraction and stabilise the suture lines of the vaginal epithelium overlying the mesh. Earlier studies reported on the use of heavyweight meshes that are now widely considered inappropriate for use in prolapse surgery.

The results of surgery described in this study compare favourably with the results of abdominal sacral colpopexy and vaginal sacrospinous fixation.^12^ Studies reporting on the use of mesh placed vaginally to treat prolapse reported success rates of 94–100%.^5^,^11^ Both studies reported only anatomical outcomes of the repaired compartments and ignored new prolapses occurring at nonrepaired sites. By adopting a similar methodology, the anatomical success rate in our study is 94%. This study reports encouraging outcomes with the surgery described. Further clinical studies, including comparative studies, are required to establish the role of this surgery.
